# Genetic Determinants of Neurobehavioral Responses to Caffeine Administration during Sleep Deprivation: A Randomized, Cross Over Study (NCT03859882)

**DOI:** 10.3390/genes12040555

**Published:** 2021-04-10

**Authors:** Mégane Erblang, Fabien Sauvet, Catherine Drogou, Michaël Quiquempoix, Pascal Van Beers, Mathias Guillard, Arnaud Rabat, Aurélie Trignol, Cyprien Bourrilhon, Marie-Claire Erkel, Damien Léger, Claire Thomas, Danielle Gomez-Merino, Mounir Chennaoui

**Affiliations:** 1 Institut de Recherche Biomédicale des Armées (IRBA), 91190 Brétigny sur Orge, France; megane.erblang@gmail.com (M.E.); catherine.drogou@gmail.com (C.D.); michael.quiquempoix@gmail.com (M.Q.); pvanbeers@gmail.com (P.V.B.); mathias.guillard@gmail.com (M.G.); arnaud.rabat.irba@gmail.com (A.R.); aurelie.trignol@intradef.gouv.fr (A.T.); cyprien.bourrilhon@intradef.gouv.fr (C.B.); marieclaire.erkel@gmail.com (M.-C.E.); dangomez51@gmail.com (D.G.-M.); mounirchennaoui@gmail.com (M.C.); 2 EA VIFASOM (EA 7330 Vigilance, Fatigue, Sommeil et Santé Publique), Université de Paris, 75004 Paris, France; damien.leger@aphp.fr; 3 LBEPS, Univ Evry, IRBA, Université Paris Saclay, 91025 Evry, France; claire.thomas@univ-evry.fr; 4 APHP, Hôtel-Dieu, Centre du sommeil et de la Vigilance, 75004 Paris, France

**Keywords:** genetics, caffeine, total sleep deprivation, PVT, pro-inflammatory cytokine, adenosine, catecholamine, gene clock

## Abstract

This study investigated whether four single nucleotide polymorphisms (SNPs) moderated caffeine effects on vigilance and performance in a double-blind and crossover total sleep deprivation (TSD) protocol in 37 subjects. In caffeine (2 × 2.5 mg/kg/24 h) or placebo-controlled condition, subjects performed a psychomotor vigilance test (PVT) and reported sleepiness every six hours (Karolinska sleepiness scale (KSS)) during TSD. EEG was also analyzed during the 09:15 PVT. Carriers of the TNF-α SNP A allele appear to be more sensitive than homozygote G/G genotype to an attenuating effect of caffeine on PVT lapses during sleep deprivation only because they seem more degraded, but they do not perform better as a result. The A allele carriers of COMT were also more degraded and sensitive to caffeine than G/G genotype after 20 h of sleep deprivation, but not after 26 and 32 h. Regarding PVT reaction time, ADORA2A influences the TSD effect but not caffeine, and PER3 modulates only the caffeine effect. Higher EEG theta activity related to sleep deprivation was observed in mutated TNF-α, PER3, and COMT carriers, in the placebo condition particularly. In conclusion, there are genetic influences on neurobehavioral impairments related to TSD that appear to be attenuated by caffeine administration. (NCT03859882).

## 1. Introduction

Several professions are exposed to prolonged wakefulness resulting in an increased risk of accident [1]. Many studies have shown that total sleep deprivation (TSD) increased daytime sleepiness and decreased sustained attention [2,3]. It is well established that during sleep loss, the most consistent parameter affected is sustained attention, which is in most research, assessed through the Psychomotor vigilance task (PVT) [4,5].

Caffeine is the major worldwide most available stimulant, used by professionals to counteract sleep loss-related neurobehavioral impairment and was shown to significantly increase alertness and decrease subjective sleepiness [6]. From a physiological point of view, caffeine globally promotes wakefulness by unselectively antagonizing adenosine receptors, particularly A2A, in the brain regions, and helps to restore cognitive function after TSD, often by preventing a decrease in sustained attention [7]. Accumulating evidence suggests that among the receptors responsible for sleep induction, the role of excitatory adenosine A2 receptor (A2A) is predominant in sleep regulation, whereas inhibitory A1 contributes to sleep induction in a region-dependent manner, but may not be absolutely necessary for sleep homeostasis. The arousal effect of caffeine has been found mediated by A2A receptors [7].

In laboratory studies on sleep deprivation, caffeine administration limited the cognitive impairment on sustained attention, with doses ranging from 200 to 600 mg [8]. However, doses of 600 mg of caffeine can cause various side effects such as abdominal pain, nausea, jitteriness, tachycardia, and tremor [8]. In fact, caffeine doses ranging from 25–300 mg (0.3–4 mg/kg) have been found sufficient enough to have a beneficial effect on PVT performance by decreasing the lapses number and improving response speed during TSD [9,10]. However, in these studies, the authors generally included non-caffeine consumers or required caffeine withdrawal prior to sleep deprivation, whereas it would probably have been preferable to require one to two months of caffeine withdrawal among regular consumers to create a “non-consumer profile” [11]. Few studies have thus been carried out with no change in habitual caffeine consumption.

Moreover, inter-individual differences exist in cognitive responses to sleep deprivation and caffeine consumption/administration based on specific genetic polymorphisms and depending on sleep and circadian influences [12]. Many studies showed the impact of genetic polymorphisms on cognitive responses to sleep loss establishing vulnerable or resilient profiles to sleep deprivation [12,13]. The circadian clock gene PERIOD3 (PER3) polymorphism determined sustained attention, subjective sleepiness [14], and working memory [15]. Other genetic polymorphisms are implicated in the inter-individual vulnerability to neurobehavioral impairment related to sleep loss such as the rs1800629 single nucleotide polymorphism (SNP) of the pro-inflammatory cytokine TNF-α [16,17]. During TSD, G/G (ancestral) allele carriers performed more lapses compared to A (mutated) allele carriers [16] with faster responses for the A allele carriers [17]. Catechol-O-methyl transferase gene (COMT), the main enzyme degrading catecholamines, is associated with neurobehavioral vulnerability to sleep deprivation [13,17] and with differentiation in sleep duration [18].

Regarding genetic interindividual vulnerability to sleep loss and/or caffeine efficiency, acute caffeine administration or habitual consumption have been studied. The most studied rs5751876 ADORA2A (gene of A2A receptor, formally designated as 1976C/T or 1083C/T) was found associated with habitual caffeine consumption [19]. This polymorphism contributed also to individual sensitivity to the effects of habitual caffeine consumption on sleep [19,20] and is associated with the anxiogenic response to caffeine [21,22]. The Bodenman et al. study [23] is the only one demonstrating that the haplotype HT4 carriers of ADORA2A performed faster on the PVT than carriers of non-HT4 haplotype alleles during TSD, and caffeine failed to counteract the PVT impairment and the rebound of slow-wave activity (SWA) in recovery sleep.

Despite the many laboratory studies on inter-individual genetic vulnerability in cognitive responses to TSD, there are little data available on the influence of combined polymorphisms and the effects of caffeine administration. Satterfield et al. [16] evidenced that it is particularly the SNP rs1800629 of TNF-α that determines a level of resilience to psychomotor vigilance performance impairment due to TSD, whereas no significant association was found with ADORA2A, PER3, TLR4, and a gene polymorphism located in the MHC nearby that of TNFα, DQB1*0602. The same group recently showed no interaction of TNFα genotype with the beneficial effect of caffeine (200 or 300 mg) on performance during TSD [24].

Our study aimed to evaluate the influence of four SNPs and caffeine effects on PVT psychomotor vigilance and subjective sleepiness during 38 h of prolonged wakefulness in healthy subjects. We also performed EEG spectral analysis during the 09:15 PVT tests at 2-h and 26-h of prolonged wakefulness [25]. According to the literature, the four SNPs selected for their involvement in cognitive vulnerability to sleep loss and/or sensitivity to the effects of caffeine are TNF-α (rs1800629), ADORA2A (rs5751876), PER3 (rs228697), and COMT (rs4680). This choice illustrated that individual cognitive vulnerability to TSD may be dependent on the homeostatic and circadian systems with a significant impact on the inflammatory and catecholaminergic profiles. In this study, we included regular caffeine consumers (247 ± 23 mg per day) to have a population as close as possible to the French population [9]. We hypothesized that selected genetic polymorphisms should modulate the effects of caffeine on neurobehavioral and neurophysiological markers of sleep deprivation.

## 2. Materials and Methods

### 2.1. Participants

38 subjects, aged between 22 and 52 years, were included. The study received the agreement of the Cochin—CPP Ile de France IV (Paris) Ethics Committee and of the French National Agency for Medicines and Health Products Safety (ANSM) (Ile de France IV) (ID-RCB: 2017-A02793-50 (CPP IDF IV), and was conducted according to the principles expressed in the Declaration of Helsinki of 1975, as revised in 2001. All of the participants gave their informed written consent.

To have at least a minimum of 12 participants per group, the last 12 subjects were selected specifically according to their genotypes, giving priority to the TNF-α and ADORA2A genotypes. Subjects were free from medical, psychiatric, and sleep disorders (insomnia, sleep apneas, hypersomnia, or parasomnia). Other exclusion criteria [3] included physical or mental health troubles based on (I) Hospital Anxiety and Depression scale [26] (HAD) ≥ 16, (II) significant medical history, (III) Epworth Sleepiness Scale [27] (ESS) > 10, (IV) Pittsburg sleep quality index [28] (PSQI) > 8, (V) morningness-eveningness questionnaire [29] <31 or >69, (VI) habitual time in bed per night < 6 h. Subjects were prohibited from using medications with sleep-related side effects and illicit drugs, or from abusing alcohol. Subjects did not travel between time zones within 7 days and did not work in shifts in the 2 weeks prior to this study. Subjects were required to complete a sleep/wake schedule for the week prior to the study.

### 2.2. Study Design and Testing Conditions

This study has been conducted in the sleep laboratory of the Armed forces biomedical research institute (IRBA), Brétigny Sur Orge, 91190, France. Ambient temperature was controlled and maintained at 22 ± 1 °C during all the experiments. The brightness of the lighting has been maintained between 150–200 lux during the awaking periods and lights were off during sleep periods. Meals and caloric intake were standardized for all subjects (2600 kcal/day).

**Figure 1:**
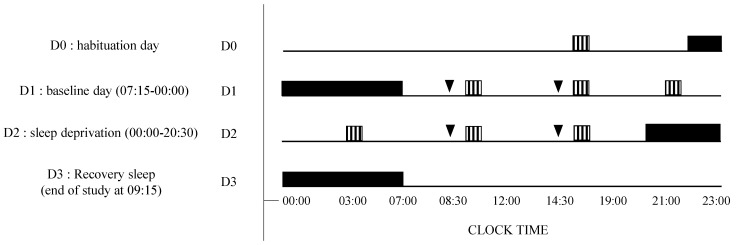
Experimental design. D0 is the habituation day, D1 is the baseline day, D2 is the day of prolonged wakefulness (i.e., sleep deprivation, between 00:00–20:30) and D3 is the recovery sleep and end of the study. Night sleep are the black bars, awaking periods black line, cognitive tests are the striped bars, and caffeine or placebo intake are the black arrows. Visual Analogic Scales (VAS), Karolinska Sleepiness Scale (KSS), and Psychomotor Vigilance Task (PVT) have been assessed at D1: 09:15, 15:15, 21:15 and D2: 03:15, 09:15, 15:15). 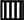 VAS, KSS, PVT; 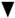 Placebo or caffeine (2.5 mg/kg) treatment (at D1 and D2: 08:30, 14:30).

Subjects remained inside the laboratory for 3 consecutive days. The experimental protocol included (I) a habituation/training day (D0), (II) a baseline day (D1) beginning at 07:15 until 00:00, (III) a total sleep deprivation (TSD) day beginning on D2 00:00 until 20:30 (i.e., 38 h of continuous wakefulness), and (IV) a recovery night until the end of the study (09:00 on D3) (Figure 1).

Subjects were not allowed to practice exercise, taking tobacco, alcohol, or other psychoactive substances during the study. They were under visual surveillance of research staff members. In addition, we used wrist actigraphy to check that the subjects stayed awake during the 38-h continuous wakefulness period. When they were not engaged in testing, meals, or sleep periods, participants were allowed to read, watch videos, or speak with other participants or staff members and playing games, following a pre-established program.

### 2.3. Caffeine Administration

In this double-blind, crossover, and placebo-controlled caffeine administration study, subjects participated in two conditions (i.e., caffeine or placebo, administered twice on D1 and D2). (Figure 1).

Each participant received, for the caffeine condition, 2.5 mg/kg body weight of caffeine powder mixed in the decaffeinated beverage. Placebo was a decaffeinated beverage with the same bitterness, smell, and taste. The caffeine powders were pre-measured by the project supervisor. This amount of caffeine powder was chosen for its enhancing properties on attention in sleep-deprived conditions (2.5–8 mg/kg of caffeine) [30]. The beverage was administered at 08:30 and 14:30 (1.5 and 7.5 h of prolonged wakefulness) on D1, and at 08:30 and 14:30 (25.5 and 31.5 h of prolonged wakefulness) on D2. For each administration, tests were performed after 45 min of ingestion.

### 2.4. Measurements

Throughout the experiment, subjects completed two tasks on a desktop computer every 6 h during D1 and D2. The psychomotor vigilance task (PVT) and Karolinska sleepiness scale (KSS) were performed at 09:15, 15:15, 21:15 on D1, and at 03:15, 09:15, and 15:15 on D2 (Figure 1). The lapse number and reaction time parameters in PVT were shown to increase the likelihood of finding differences between sleep-deprived and alert states with small sample sizes [4].

EEG was registered during the 10 min PVT to evaluate theta and alpha power at 2 h (D1, 09:15) and at 26 h (D2, 09:15) of prolonged wakefulness. All subjects had a systematic habituation period for tests at D0 (habituation/training day) to reduce a learning bias during the first set of tests. Before each PVT, the visual analog scale (VAS) and KSS were self-reported. Vital signs such as blood pressure and heart rate were assessed after each PVT.

#### 2.4.1. Psychomotor Vigilance Task (PVT) for Sustained Attention

We used a computer-based version of the 10-min PVT [31]. Subjects were instructed to respond, by clicking the left mouse button, as soon as the visual stimulus appeared (incrementing millisecond counter) without making false starts. The inter-stimulus interval was randomized between 2 and 10 s. The reaction time (RT) is quantified in milliseconds for a 1 s period and the response was regarded valid if RT was ≥ 100 ms. Results are expressed as the number of lapses (RTs > 500 ms) and speed [4].

#### 2.4.2. Karolinska Sleeping Scale (KSS)

The KSS is a subjective scale used to grade the subject sleepiness from 1 to 9, 1 is for “extremely alert” and 9 for “extremely sleepy” [32]. The informatic version used in this study enables the subject to choose out of the nine given options. The KSS was carried out before each PVT test.

#### 2.4.3. Visual Analogic Scales (VAS)

Spontaneous adverse events (AEs) were reported on a 100 mm scale on a computer with to each end “not at all” and “a lot”. Six symptoms were evaluated because of their association with caffeine consumption [8]: headache, nausea, stomach pain, and fatigue. When subjects pointed above 20 mm, it was considered as an AE pattern. If an AE was observed during the placebo condition, it is considered to be related to sleep deprivation; if it was observed during caffeine condition, it may be related to sleep deprivation and/or caffeine consumption. As well as the KSS, VAS was administered before each PVT. Only adverse events >20 mm on the 100 mm scale are reported.

#### 2.4.4. EEG Recording during PVT

##### EEG Procedure

EEG was recorded at 19 scalp sites according to the international 10–20 system (Fp1, Fp2, F7, F3, Fz, F4, F8, T7, C3, Cz, C4, T8, P7, P3, Pz, P4, P8, O1, O2) with a Siesta^®^ 802 (Compumedics Limited, Victoria, Australia). The EEG was recorded continuously at a sampling rate of 512 Hz and referenced with a common average. Electrodes were interfaced with the scalp using EC2 gel (Grass Technologies, Astro-Med, Inc., West Warwick, RI, USA), and impedances were kept below 10 kOhm during the whole session. The EEG was installed approximately 20–30 min before the PVT.

##### EEG Analysis

EEG spectral analysis data were analyzed in Matlab (Mathworks, Natick, MA, USA) with Fieldtrip toolbox and custom codes [33]. Data were band-stop filtered between 48 and 52 Hz to remove electrical noise, high-pass filtered above 0.1 Hz, and locally detrended. Blink artifacts were removed by computing an Independent Component Analysis (ICA, Fieldtrip), and movement artifacts were removed by visual inspection. Bad electrodes were systematically rejected, and if more than 3 (out of 19) electrodes were removed, the subject was excluded from EEG analysis. EEG theta power (4–8 Hz) was assessed by using continuous Morlet wavelets transform. Regions of interest (ROI) represent the mean of grand averaged (all subjects) theta power over frontal (Fp1, Fp2, F7, F3, Fz, F4, F8) and centrotemporal (T7, C3, Cz, C4, T8) regions.

#### 2.4.5. Genotyping

Genotyping for the rs5751876 ADORA2A, rs1800629 TNF-α, rs4680 COMT, and rs228697 PER3 were performed on blood cells using the LAMP-MC method [34]. This method has been applied to complex biological matrices, such as whole blood and saliva, without prior DNA extraction.

Blood cells samples were collected on EDTA and were aliquoted in sterile microtubes and stored at −20 °C until LAMP-MC analysis. The LAMP-MC genotyping assays were realized by use of the customized Human Sleep Deprivation Combo kit (Cat#LCSDC-LP-24, LaCAR MDX, Liège, Belgium). Positive control and negative control were supplied for each SNP. LAMP-MC consists of a lysis of cells followed by the amplification of the target sequence at a constant temperature around 65 °C using simultaneously three sets of primers, a polymerase with high strand displacement activity in addition to a replication activity and a fluorophore-labeled probe. Detection of homozygous wild, heterozygous and homozygous mutant genotype is performed by melting curve analysis after amplification [34].

### 2.5. Statistical Analysis

Statistical analyses were computed using R-studio (V 0.99.902-2009–2016 RStudio, Inc., Vienna, Austria). Values were expressed as mean ± SEM. If a group of homozygous mutation counts less than 6 members, heterozygous and homozygous are grouped for statistical analysis and graphic. However, when the subject group carrying a homozygous ancestral genotype counted less than 6 members, we chose to leave it to constitute comparable groups. A T-test with R studio was used to compare individual characteristics of subjects according to their genotype for each SNPs, and to compare EEG theta-to-alpha ratio between genotypes in the frontal and centrotemporal brain regions. Results for PVT speed and lapses and KSS were analyzed using a three-way mixed-effect analysis of variance (ANOVA) including fixed effects for genetic polymorphism (non-repeated measures), treatment (caffeine or placebo, repeated measures), and awakening duration (repeated measures).

One-way ANOVA was used to test for differences between genotypes in age, caffeine habits, total sleep time, and race/ethnicity distributions. Secondary analyses (3-way ANOVAs: treatment, awakening, and the studied factor) also controlled the lack of interaction with age, gender, caffeine habits, and total sleep time for reaction time and lapses.

A Tukey post-hoc test was used to identify differences between genotypes for awakening and treatment conditions. For the awakening effect, all points were compared to the 9:15 baseline. P-value has been corrected for multiple comparisons using the Bonferroni method. A correlation analysis was used to study the relationships between EEG alpha and theta power during the PVT testing after sleep deprivation (D2 day) using the Pearson-correlation. Changes in EEG spectral activity were analyzed using the Z-scored FFT method. The application of the mathematical Gaussian curve, i.e., “bell curve”, via the estimation of probabilities using the auto- and cross-spectrum of the EEGs is defined as the Z-scored FFT method [35].

## 3. Results

### 3.1. Participants Characteristics

38 subjects participated in this study (Figure 1). We excluded 1 participant because of an important adverse effect after caffeine intake. Finally, a total of 37 healthy subjects (33.5 ± 1.3 years) followed the protocol including 21 females and 16 males. Subjects’ average daily caffeine consumption was 250 ± 32 mg (mean ± SEM). The mean BMI was 23.5 ± 0.6 for men and 23.0 ± 0.6 for women. The mean weekly exercise duration was 3.1 ± 0.4 h. The mean daily total sleep time was 7.2 ± 0.2 h and the sleepiness score was 6.8 ± 0.6. Chronotype was distributed this way: 20 subjects were of intermediate chronotype, 16 were of the morning with 4 being clearly of the morning, and 1 was of the evening.

### 3.2. Adverse Events (AEs)

With the exception of the excluded participant, no serious AEs (>20 mm on the 100 mm scale) were reported. The participant reported nausea and vomiting. For the headache AE, significant treatment (TRT) is observed (*p* < 0.01) with more headache reported during the placebo compared to caffeine condition. For the nausea symptom and abdominal pain, a significant awakening (TSD) effect is observed (*p* < 0.01, respectively) with increases as the waking time increases. For the fatigue AE, significant TSD and TRT (*p* < 0.01, respectively) effects were observed but without interaction. The fatigue increased with TSD in the 2 conditions but less in caffeine. No significant effect of the 4 SNPs was found.

### 3.3. Genotypes Repartition

Participant genotypes and their corresponding characteristics are summarized in Table 1. Genetic variations for the rs1800629 TNF-α, rs5751876 ADORA2A, rs4680 COMT, and rs228697 PER3 are G > A, C > T, G > A and C > G respectively. In addition, ADORA2A T allele carriers have daily caffeine consumption higher than C/C genotype carriers. The frequencies for TNF-α genotypes were different from the 1000 Genomes project database population because, after reaching three-quarters of the study, we chose to select participants based on their genetic profile to balance specific genotypes that are particularly rare in the population. We chose to prioritize TNF-α and ADORA2A polymorphisms in regard to the scientific literature. As the mutated homozygous or heterozygous alleles were extremely rare for some polymorphisms (TNF-α A/A, ADORA2A T/T, and PER3 C/G), mutated homozygous and heterozygous allele were combined. Their aggregation did not modify their significant effect on the parameters evaluated.

There were no significant differences between the genotypes (Table 1) for age, gender, and TST (ANOVA results not shown). Concerning habitual caffeine consumption, we observed a significant difference between group only for ADORA2A (F_1, 81_ = 1.42, *p* = 0.04). No differences were observed for the other polymorphisms.

### 3.4. Genetics and Inter-Individual Vulnerability to Caffeine Administration during Sleep Deprivation

The ANOVA statistical analysis of the data depicted significant awakening (TSD) and treatment (TRT) effects for the 4 SNPs on PVT number of lapses, speed, and KSS score (*p* < 0.01 for all) but no significant effect of SNP (Supplementary Table S1). An ANOVA interaction between TSD × TRT was observed for the PVT lapses only (*p* < 0.01) (Table 2).

#### 3.4.1. Awakening (Total Sleep Deprivation—TSD) and Caffeine (Treatment—TRT) Effects

**Figure 2:**
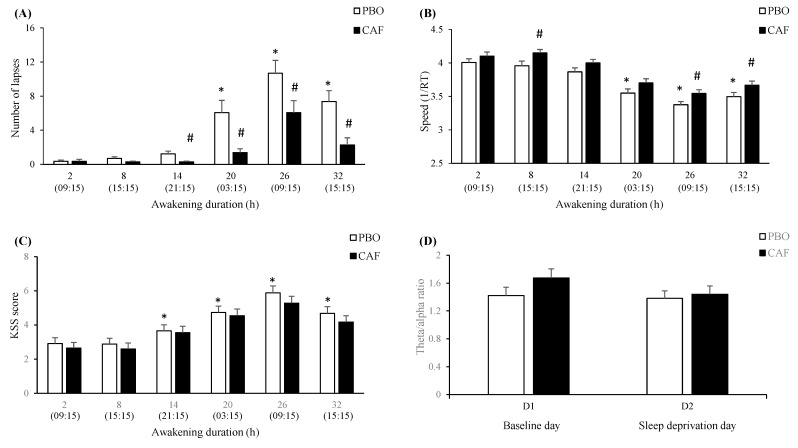
Mean PVT performance over 38-h of prolonged wakefulness for a number of lapses (**A**), speed (**B**), KSS score (**C**), and the EEG theta-to-alpha ratio (**D**) during PVT in the centrotemporal brain region at 09:15 on D1 (baseline) and D2 (sleep deprivation) days as a function of placebo (PBO) or caffeine (CAF) condition. Values are mean ± SEM. * difference between baseline and continuous wakefulness, # between PBO and CAF conditions.

Compared to 9:15 (at day 1), there are significant increases of lapses and KSS score from 14-h to 32-h of awakening (Figure 2A–C). In caffeine (CAF) compared to placebo (PBO) condition, the number of lapses was significantly lower from 14-h to 32-h of awakening. Regarding speed, it was significantly higher in CAF compared to PBO condition at 8-h, 26-h, and 32-h of awakening. For the KSS score, no significant difference was observed between CAF and PBO conditions (Figure 2C). There was a significant interaction between awakening (TSD) and treatment (TRT) for the EEG theta-to-alpha ratio in the centrotemporal brain region without significant post-hoc differences (Supplementary Table S2 and Figure 2D). Interindividual differences in performance impairment during TSD were not predicted by age (F_1, 420_ = 0.17, *p* = 0.86) or gender (F_1, 420_ = 1.11, *p* = 0.29) and no interaction was observed with treatment or awakening.

#### 3.4.2. SNPs Effects

**Figure 3:**
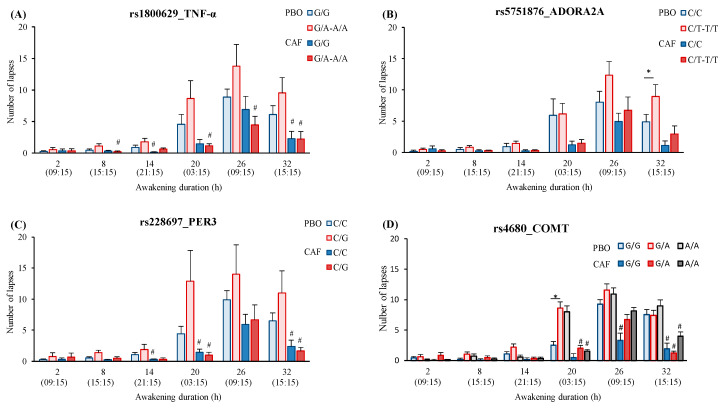
PVT number of lapses across consecutive 6-h intervals of awakening according to polymorphisms of TNF-α (**A**), ADORA2A (**B**), PER3 (**C**), and COMT (**D**) in placebo (PBO) and caffeine (CAF) conditions. Caffeine was consumed 45-min before PVT after 2-h, 8-h, 26-h, and 32-h of prolonged wakefulness. * is a SNP difference (*p* < 0.05), # is a treatment difference (*p* < 0.05) for one genotype.

**Figure 4:**
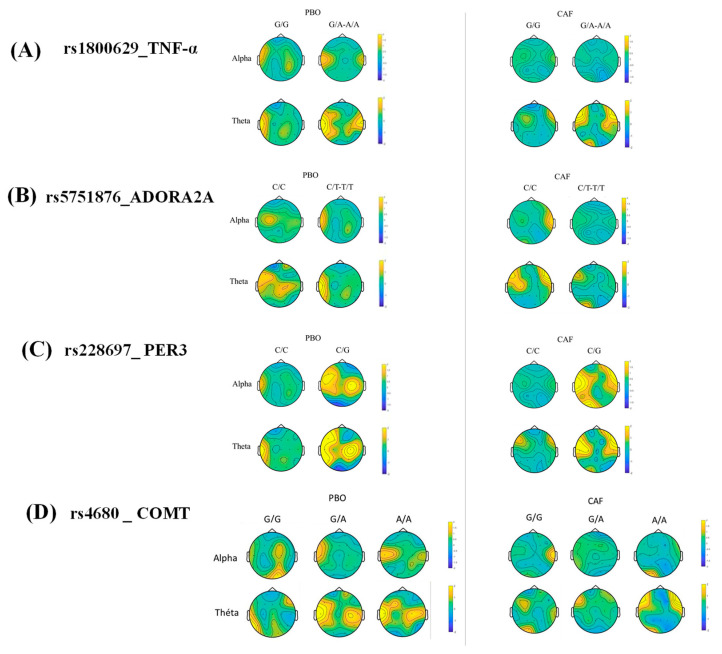
EEG theta and alpha normalized power on D2 day (sleep deprivation) relative to D1 day (baseline) (z-score) on brain scalps during PVT at 09:15 according to polymorphisms of TNF-α (**A**), ADORA2A (**B**) and PER3 (**C**), and COMT (**D**) in placebo (PBO) and caffeine (CAF) conditions. Caffeine was consumed 45-min before PVT at D1 and D2.

For the TNF-α SNP effect on PVT lapses, there is a significant 2-way interaction with TRT and significant 3-way interaction with TRT and TSD (Table 2). The post-hoc analysis showed no statistical differences between genotypes at any time in TSD (Figure 3A). However, there is a CAF effect with a significantly lower number of lapses (compared with PBO condition) in A allele carriers at 8-h, 20-h, 26-h, and 32-h, while this is present at 14-h and 32-h in G/G genotype carriers (Figure 3A). Regarding speed, a significant interaction of TNF-α SNP with TSD is observed but without significant post-hoc, and no significant interaction with TRT (Table 2, Supplementary Figure S1A). Concerning the KSS score, no significant interaction of TNF-α SNP was observed neither with TSD nor with TRT (Table 2). Finally, the EEG spectral analysis on brain scalps showed higher theta activity at D2 day relative to D1 in A allele carriers in PBO and CAF conditions (Figure 4A) (9 subjects were G/A, 3 were A/A, and 19 were G/G). The ANOVA analysis on theta-to-alpha ratio in response to sleep deprivation (D2 and D1 days) in the centrotemporal region showed interaction of TNF-α SNP with TSD and interaction between TSD and TRT (Supplementary Table S2). The post-hoc analysis showed a higher theta-to-alpha ratio in A allele carriers compared to G/G genotype carriers in the PBO condition (*p* < 0.05) but no significant difference at D1 or D2 between PBO and CAF conditions.

For the ADORA2A SNP effect on PVT lapses, there is a significant interaction with TSD (*p* < 0.02), but not with TRT (Table 2). Post-hoc analysis showed higher lapses in T allele carriers compared with C/C genotype carriers at 32-h of awakening (Figure 3B). With respect to the effect of ADORA2A SNP on speed or KSS, there is no significant ANOVA interaction (Table 2). For speed, there was no significant difference between genotype carriers during TSD (Supplementary Figure S1B). Finally, the EEG spectral analysis on brain scalps showed no genotype-related difference on the theta activity either in PBO or CAF condition (Figure 4B) (16 subjects were C/T, 2 were T/T, and 13 were C/C). The ANOVA analysis on theta-to-alpha ratio in response to sleep deprivation (D2 and D1 days) in the frontal region showed a significant interaction of ADORA2A SNP with TSD (Supplementary Table S2). without significant post-hoc. In the centrotemporal region, there is no significant interaction of ADORA2A SNP with TSD, but the interaction between TSD and TRT (Supplementary Table S2), without significant post-hoc.

For the PER3 SNP (rs228697) effect on PVT, there is significant 2-way interaction with TRT for lapse number and speed, but no interaction with TSD (*p* < 0.08 and *p* = 0.82, respectively) (Table 2). In the CAF condition, there is a significantly lower number of lapses (compared with PBO condition) in G allele carriers at 20-h, and 32-h of awakening and this is present at 14-h, 20-h, and 32-h in C/C genotype carriers (Figure 3C). Concerning the speed, it was higher in the CAF condition (compared with PBO condition) in C/C genotype carriers at 26-h of awakening, while no significance was present for G allele carriers (Supplementary Figure S1C). Concerning the KSS score, the PER3 SNP has no significant effect nor interaction with TSD or TRT (Supplementary Tables S1 and S2). Finally, the EEG spectral analysis on brain scalps showed higher alpha and theta activity in G allele carriers in PBO and CAF conditions (Figure 4C) (6 were C/G and 25 subjects were C/C). The ANOVA analysis on theta-to-alpha ratio in response to sleep deprivation (D2 and D1 days) in the frontal and centrotemporal regions showed no significant interaction of PER3 SNP with TSD, nor interaction between TSD and TRT (Supplementary Table S2).

No significant COMT SNP (rs4680) main effect was observed on PVT (lapses and speed) and KSS (Supplementary Table S1), but a significant 3-way interaction is present with TRT and TSD on PVT lapse number (Table 2). After 20-h of awakening, A allele carriers performed worse than ancestral G/G with a beneficial effect of caffeine (Figure 3D). Finally, the EEG spectral analysis on brain scalps showed higher theta activity in A allele carriers in the PBO condition only (Figure 4D) (14 subjects were G/A, 7 were A/A, and 10 were G/G). The ANOVA analysis on theta-to-alpha ratio in response to sleep deprivation (D2 and D1 days) in the frontal and centrotemporal regions showed no significant interaction of COMT SNP with TSD nor TRT, nor the interaction between TSD and TRT (Supplementary Table S2).

Thirty-one subjects were analyzed because of electrodes removed or poor signal quality and numerous blinks on 6 subjects. Significance was considered at >+1.96 or >−1.96.

### 3.5. Correlation Analysis between the Lapses Number and EEG Alpha and Theta Power during the PVT Testing after Sleep Deprivation (D2 Day) in the Centro-Temporal Brain Region

**Figure 5:**
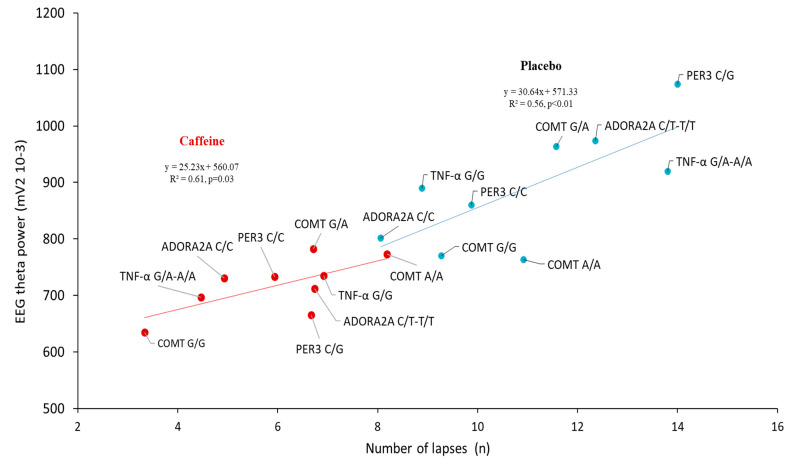
Correlation analysis at 26-h of prolonged wakefulness (D2 day at 09:15) between EEG theta power in the centrotemporal brain region and the number of lapses during the PVT for all genetic polymorphisms in placebo (blue circle, grey line) and caffeine (red circle, black line) conditions.

For the 4 SNPs, significant positive correlations were found between theta power and the number of lapses in the caffeine and placebo conditions (Figure 5). In comparison for the alpha power and number of lapses, no significant correlation was observed (*p* = 0.50 and *p* = 0.20 in placebo and caffeine conditions).

## 4. Discussion

Our study demonstrated for the first time, in a relevant number of subjects, that genes can influence the levels of psychomotor vigilance impairments associated with total sleep deprivation (i.e., corresponding to 38-h of prolonged awakening) and their modulation by caffeine administration. Caffeine seems to attenuate the genetic influence on vigilance impairments. We selected four SNPs that have been previously identified as potential genetic mechanisms of vulnerability to sleep loss and/or caffeine intake: the proinflammatory cytokine TNF-α involved in the regulation of immune cells, ADORA2A the adenosine A2A receptor-related gene, PER3 the circadian gene, and COMT the gene for catechol-O-methyltransferase (COMT), an enzyme critical for the breakdown of catecholamines [12,13,14,16,19,23].

Not surprisingly, prolonged wakefulness impaired sustained attention and sleepiness, and caffeine administration improved the neurobehavioral consequences of total sleep deprivation (TSD) with minimal adverse effects [2,5,10,36]. Our results also evidenced significant awakening (total sleep deprivation—TSD) and caffeine (treatment—TRT) effects on PVT parameters and sleepiness score, with interaction on the PVT lapses number only. We also described awakening and TRT main effects and interaction on the EEG theta-to-alpha power ratio during the 09:15 PVT testing in the centrotemporal brain region as previously described [37,38,39]. In this brain region, the EEG theta power and the number of lapses are significantly correlated at the D2 day (i.e., corresponding to 26 h of prolonged wakefulness) for all polymorphisms in placebo and caffeine conditions, underlying the well-established arousing effect of caffeine. At last, there are also no serious side effects of awakening or caffeine in this study.

The common TNF-α variant G-308A (rs1800629) (GRCh38 38.1/141 reference genome, on chromosome 6) is located on 31,575,254 position. It involves guanine to adenine substitution at position 308 in the promoter region of the gene. It was shown to alter TNF-α gene transcriptional activity [40] and increase TNF-α production in whole blood cell culture in healthy subjects [41]. TNF-α has been proposed as a cause of cognitive instability, observed by an increase in PVT attention deficits, for example, by inducing a local sleep-type state [42], and we have previously shown an increase in TNF-α levels and PVT lapse deficits after 36 h of continuous wakefulness in healthy men [3,43].

In our study, TNF-α polymorphism is significant in a three-way interaction with awakening (TSD) and caffeine (TRT) on PVT lapses only. Post-hoc analysis showed no statistical difference between genotypes on any of the PVTs criteria during TSD, regardless of treatment condition. However, there was significance between genotypes for the effects of caffeine during TSD. The A allele carriers experienced an alleviating caffeine effect on lapses at four PVT timing of TSD while this effect was present at two-timing in the G/G genotype carriers. This suggests that carriers of allele A benefit more from the stimulating effect of caffeine than G/G only because they seem more degraded, but their performance is not higher. Regarding PVT speed, the TNF-α polymorphism significantly interacts with awakening but without significant post-hoc, and not with caffeine.

Our results on PVT lapses partly contradict those of Satterfield et al. [16] who show that carriers of TNF-α G/G genotype are more vulnerable than A allele carriers to sleep deprivation. In addition, they did not find interaction with caffeine administration (provided in the chewing gum) but with a small group of subjects (four carrying the A allele and eight being homozygote G/G) carriers the [24]. In their first study [16], subjects had to abstain from caffeine only for the week prior to each laboratory experiment, whereas our subjects maintained their usual caffeine consumption until the experiment to be as close as possible to real-life conditions. Moreover, we observed no significant difference in habitual caffeine consumption between TNF-α genotypes in our subjects. Thus, in Satterfield et al. [16], we can suggest that regular caffeine consumers may have experienced withdrawal symptoms [11] that may have altered their neurobehavioral response to sleep deprivation [44]. The amount of variance in PVT lapses being only 6.4% for TNF-α polymorphism [16], the effect of habitual caffeine consumption and withdrawal may have been more pronounced on responses to sleep deprivation than variability induced by TNF-α polymorphism. Another explanation for the difference of our results with Satterfield et al. [16] regarding genotype vulnerability to sleep deprivation may be the genotyping method. In our study, this genotyping was done in duplicate with two methods, the LAMP-MC, and the reference Taqman, and the concordance was high [34]. The genotype frequencies in our study are additionally compared and consistent with those of the 1000 Genomes Project [45].

With respect to subjective sleepiness, we did not find significant interaction of TNF-α polymorphism neither with awakening nor with caffeine treatment and caffeine administration alleviating its increase induced by the duration of wakefulness whichever polymorphism.

The EEG spectral analysis on brain scalps showed higher theta activity in A allele carriers compared to G/G carriers in the placebo and caffeine conditions. In addition, the theta-to-alpha ratio in response to sleep deprivation (i.e., D2 day) is higher in the centrotemporal region in the placebo condition only. This result underlined the interest in examining theta and alpha EEG frequencies, during repetitive PVT testing. It also illustrates the sensitivity of TNF-α A allele carriers to sleep deprivation-related increase of sleepiness during the PVT testing. Our findings confirmed that an increase in theta activity may be the principal EEG basis of the post-sleep deprivation lapses impairments as previously described [38]. This is strengthened by the positive correlation between the EEG theta frequency in the centrotemporal region and the lapse number after 24 h of sleep deprivation for the four polymorphisms and either the placebo or caffeine conditions. The two linear regression curves illustrate how carriers of the TNF-α A allele benefit from the effects of caffeine on lapse number and theta activity.

To conclude on the interaction of TNF-α polymorphism with the beneficial effect of caffeine during TSD, we showed that A allele carriers appear to be more sensitive than carriers of the ancestral G/G genotype. This is only because they seem more degraded, but they do not perform better as a result. As caffeine concentrations relevant for human consumption have been shown to systematically suppress the production of TNF-α in human blood [46], it would be prudent to consider the genetic of TNF-α when faced with sleep deprivation. Our results also highlight the need to evaluate the TNF-α protein expression in blood-related to subgroups of TNF-α rs1800629 genotypes carriers and the corresponding changes in response to caffeine and sleep deprivation.

The genetic influence of the gene ADORA2A, coding for A2A receptor, on neurobehavioral performance and sleep EEG during sleep deprivation combined with caffeine administration has only been described through haplotype analysis [23]. The rs5751876 SNP is a synonymous variant (i.e., it does not cause an amino acid change in the encoded protein) located on the exon 4 position on Chr. 22q11.23, presumed in the past to exert no functional effect. However, evidence has emerged that synonymous substitutions can have functional consequences affecting various steps of protein biosynthesis resulting in changes in protein abundance and structure, mechanisms including disruption or creation of splicing regulatory sites, alterations of mRNA stability, gain or loss of miRNA binding sites, and changes in translation efficiency [47]. We previously indicated increased mRNA expression of A2A receptors after total sleep deprivation in leukocytes of healthy subjects and PVT lapses impairments [3,48]. In our study and for PVT lapses only, the ADORA2A rs5751876 SNP significantly interacts with awakening but not with caffeine. We hypothesis that T allele carriers (C/T-T/T) made higher PVT lapses compared to homozygote ancestral C carriers and caffeine treatment alleviates the deficit in both allele carriers. The T allele carriers may thus have structural differences in their A2A receptors compared to the ones of ancestral C/C genotype carriers, consequently rendering them sensitive to prolonged wakefulness [47]. In any case, our results seem difficult to compare with those of Bodenmann et al. [23] who show that the haplotype called “HT4” carriers (which included rs5751876 T allele) performed faster than non-HT4 on PVT speed during total sleep deprivation, and seem resistant to caffeine counteracting effect on the waking-induced impairment of PVT performance.

Our recent previous study described that the influence of ADORA2A polymorphism on daily total sleep time is present only in subjects habitually consuming low caffeine (0–50 mg/day), with the ancestral C/C genotype carriers sleeping more than T allele carriers [19]. In this study, we suggested that C/C genotype carriers appear to be very sensitive to caffeine consumption because their daily sleep time is similar to that of T allele carriers as soon as the daily caffeine consumption exceeds 50 mg/day (one expresso per day). In the herein presented study, although homozygous C/C carriers had significantly lower habitual daily caffeine consumption than T allele carriers (168 vs. 300 mg/day, only the equivalent of one espresso), the 168 mg/day is far inferior to the caffeine administration during the experimental protocol (350 mg with two administrations). Thus, C/C carriers may, therefore, have experienced an acute effect of caffeine in reducing sleep pressure compared to T allele carriers during total sleep deprivation, making them less degraded.

Our findings evidenced no change of EEG theta activity during the PVT task after 24 h of sleep deprivation in placebo and caffeine conditions. However, the ADORA2A polymorphism interacts with an awakening for the sleep deprivation-related theta-to-alpha ratio in the frontal brain region. Considering the absence of ADORA2A polymorphism interaction with caffeine, our results seem in line with a recent study demonstrating that caffeine effect on cognitive PVT performance is influenced by the CYP1A2 genotype, the gene of the P450 enzyme which converts caffeine to its major metabolite paraxanthine, but not by the ADORA2A genotype in healthy adults performing exercise [49]. It would be interesting to have future studies on the influence of CYP1A2 polymorphism on neurobehavioral responses to sleep deprivation.

It is well known that PER3 is a circadian gene that is in a robust association with diurnal preference, mood, and anxiety levels [50,51]. It modulates sleep characteristics and mediates consequently waking performance [52]. It was shown to interact with sleep homeostasis and circadian phase during functional brain responses to an executive task [53]. In this study, the polymorphism of PER3 rs228697 is found in a significant ANOVA interaction with caffeine treatment on PVT lapses and speed. In the caffeine condition compared with placebo, the C/C genotype carriers performed fewer lapses at 14-, 20-, and 32-h of wakefulness and were faster (at 26-h), while the significance for G allele carriers was at 20-h on lapses only. This may suggest a higher effect of caffeine in homozygous C/C carriers, while the lower effect in G allele carriers may be related to their higher level of anxiety than that of C/C carriers found in a large student population [51]. In addition, for KSS, PER3 SNP did not interact with awakening and treatment respectively, while a nearly significant three-way ANOVA interaction was present with awakening and treatment (*p* = 0.06). The additional information we can provide on sleepiness indices during awakening is the higher EEG theta and alpha activity during PVT on the brain scalp of G allele carriers than in homozygous C/C in the placebo and caffeine conditions. The main findings of the influence of PER3 polymorphism on the neurobehavioral performance of healthy individuals submitted to acute sleep deprivation concerned the PER3^4/4^ and PER3^5/5^ [14], and there are no data at our knowledge on the combined influence of caffeine during total sleep deprivation. Individuals with the 5/5 genotype had greater difficulty sustaining performance across a 10 min PVT than the 4/4 group under high homeostatic sleep pressure related to 40 h prolonged wakefulness [14]. In contrast, no association was found between vulnerability to sleep loss quantified by the average of PVT lapses for PER3 polymorphism, which can be explained by the low amount of variance which (2.1%) [16]. Lo et al. [15] observed higher impairment on executive functions by acute sleep deprivation than sustained attention, and that differences between genotype and tasks in response to sleep deprivation are dependent on the circadian phase at which performance is assessed.

We also focused on PER3 rs228697 because the LAMP-MC genotyping method is much faster than genotyping performed with polymerase chain reaction (PCR) using primers described by Ebisawa et al. [54]. For our selected PER3 rs228697, variant G has been described in almost complete linkage disequilibrium with the PER3^4^ variant (rs57875989), a variable number tandem repeats polymorphism non-analyzable by loop amplification, in a large Caucasian population living in northern Italy [55]. In this study, the frequency of allelic variants at two PER3 polymorphic sites, rs57875989 (PER3^4^, PER3^5^) and rs228697 (PER3^C^, PER3^G^) were studied in buccal cells. No significant relationship was observed between PER3 rs57875989 polymorphism and diurnal preference, while a significant association was observed between the rs228697 G variant and morningness and also between the PER3^G^-PER3^4^ haplotype and morningness. With respect to the relationship between PER3 and caffeine, data are scarce, but Gamble et al. [56] demonstrated that three polymorphisms of PER3 were associated with increased caffeine consumption in night-shift nurses only and decreased likelihood of drowsiness. Studies with large groups of healthy subjects are therefore needed to extend knowledge of the influence of the polymorphism PER3 rs228697 on the effects of caffeine on neurobehavioural performance during sleep deprivation.

The catechol-O-methyltransferase (COMT) gene, the major enzyme degrading catecholamines, particularly dopamine, has also been implicated in interindividual variation in brain alpha oscillations and also in vulnerability to sleep loss [13,57]. The Val158Met polymorphism (rs4680) markedly affected enzyme activity, protein abundance, and protein stability [58]. In our study, the COMT rs4680 is in significant interaction with awakening and caffeine administration for the PVT lapse number only. In the placebo condition, the A allele carriers are significantly more degraded after 20 h of sleep deprivation than homozygous G/G ancestral carriers and responded to alleviating effect of caffeine administration. These A allele carriers exhibited higher EEG theta activity in the placebo and not caffeine condition, underlying the arousing effect of caffeine. However, with respect to PVT performance on lapses, there is no polymorphism difference after 26 and 32 h of sleep deprivation, the caffeine beneficial effect was statistically present for the three genotypes when reaching 32 h of sleep deprivation.

There are two studies of the same researchers related to COMT’s influence on neurobehavioral performance during total sleep deprivation, one on the PVT task and the other on the executive Go/NoGo task [13,17]. We did not replicate the interaction between the time awake and COMT genotypes observed on PVT reaction time [17]. The lower number of participants in our study may explain the lack of significance on the interaction of polymorphism with awakening as observed in the retrospective study of Satterfield et al. [17]. The two previous studies of this team evidenced that the influence of COMT polymorphism on neurobehavioral performances during total sleep deprivation depends on the cognitive task and is different between attentional and executive performances. Regarding the influence of COMT polymorphism on the effects of caffeine administration during acute total sleep deprivation, there are little data on our knowledge, the only one concerned the DAT1 dopamine transporter [39]. The DAT1 polymorphism was found to modulate the effects of caffeine on low-frequency brain oscillations in wakefulness and sleep, and slow waves in NREM sleep, and the rebound in SWS and EEG SWA after sleep deprivation [39]. Our results added original information regarding the COMT influence on the neurobehavioral response to total sleep deprivation and its interaction with the beneficial effect of caffeine.

It should be noted that multiple co-factors can modulate the interindividual neurobehavioral variability response to caffeine effects during prolonged wakefulness, sometimes more influential than the genetic impact. Co-factors such as age or gender influence sleep quality and EEG spectral power density as well as cognitive performance during sleep loss [59,60,61,62]. It is meaningful to notify that our sample size was small but with the advantage of limited racial and ethnic diversity.

## 5. Conclusions

In conclusion, the genetic influences on neurobehavioral impairments related to TSD appear to be attenuated by caffeine administration in healthy young subjects. The consideration of potential genetic influences in the operational performance of sleep-deprived subjects would allow the military command to individualize advice in terms of caffeine consumption, for example. Future studies could try to recruit a larger population (>100 subjects), with a more diverse profile (age, gender, lifestyle habits, caffeine consumption), and periods of awakening longer than 36-h or repeated exposition [10].

## Figures and Tables

**Table 1:** Genetic polymorphisms distribution and individual characteristics in the studied population compared to 1000 Genomes international database, an expected based on 1000 Genomes Project data on the GRCh38 reference assembly (http://www.internationalgenome.org, latest access on 04/09/2020).

Genetic Polymorphism (*Chromosome*, *Location*)	Genotypes	N (%)	1000 Genomes (%)	Age (Years)	Gender (♀, %)	Habitual Caffeine Consumption (mg/day)	TST (Hours)
**rs1800629_TNF-α**	G/G (ancestral)	24 (64.9%)	74.4%	34.8 ± 1.8	58.3%	244 ± 42	7.37 ± 0.2
(6:31.575.254)	G/A-A/A	13 (35.1%)	25.6%	31.2 ± 1.8	53.8%	261 ± 49	6.83 ± 0.3
**rs5751876_ADORA2A**	C/C (ancestral)	14 (37.8%)	37.4%	32.9 ± 1.8	57.1%	168 ± 53	7.23 ± 0.3
(22:24.441.33)	C/T—T/T	23 (62.2%)	62.6%	33.9 ± 1.8	56.5%	300 ± 36 *	7.14 ± 0.2
**rs228697_PER3**	C/C (ancestral)	31 (83.8%)	81.7%	34.4 ± 1.5	58.1%	241 ± 35	7.10 ± 0.2
(1:7.827.519)	C/G	6 (16.2%)	18.3%	29.0 ± 2.5	50.0%	296 ± 84	7.58 ± 0.3
**rs4680_COMT**	G/G (ancestral)	11 (29.7%)	26.4%	32.7 ± 1.7	72.7%	249 ± 69	6.83 ± 0.3
(22:19.963.748)	G/A	14 (37.8%)	47.1%	37.4 ± 2.1	35.7%	312 ± 47	7.62 ± 0.2
	A/A	12 (32.4%)	26.5%	30.0 ± 2.5	66.6%	178 ± 48	6.97 ± 0.3

Values are mean ± SEM. * (and bold) difference between genotypes in the same polymorphism (*p* < 0.05).

**Table 2:** ANOVA analysis of genetic polymorphism (SNPs) in interaction with awakening (TSD) and treatment (TRT) on PVT (psychomotor vigilance task) parameters (number of lapses and speed) and KSS score.

Parameters	SNPs	Awakening × Treatment	Polymorphism × Awakening	Polymorphism × Treatment	3-Way Interaction
		TSD × TRT F_5, 175_ (p)	SNP × TSD F_5, 175_ (*p*) *	SNP × TRT F_1, 35_ (*p*) **	SNP × TSD × TRT F_5, 175_ (*p*) *
**PVT Lapses**	**rs1800629_TNF-α**	**19.17 (<0.01)**	1.87 (0.17)	**6.03 (0.02)**	**4.04 (0.05)**
	**rs5751876_ADORA2A**	**19.64 (<0.01)**	**5.67 (0.02)**	0.59 (0.45)	0.53 (0.47)
	**rs228697_PER3**	**18.71 (<0.01)**	3.02 (0.08)	**10.2 (<0.01)**	2.82 (0.09)
	**rs4680_COMT**	**14.70 (<0.01)**	0.15 (0.81)	1.86 (0.15)	**5.06 (0.04)**
**PVT Speed**	**rs1800629_TNF-α**	0.74 (0.39)	**6.59 (0.01)**	1.29 (0.26)	0.27 (0.61)
	**rs5751876_ADORA2A**	0.64 (0.42)	0.68 (0.41)	0.46 (0.50)	0.00 (0.97)
	**rs228697_PER3**	0.64 (0.42)	0.05 (0.82)	**5.80 (0.02)**	0.18 (0.67)
	**rs4680_COMT**	0.74 (0.39)	0.68 (0.41)	1.60 (0.21)	0.30 (0.61)
**KSS**	**rs1800629_TNF-α**	0.94 (0.33)	1.89 (0.35)	0.75 (0.39)	0.48 (0.49)
	**rs5751876_ADORA2A**	0.87 (0.35)	2.83 (0.09)	0.76 (0.39)	0.24 (0.62)
	**rs228697_PER3**	0.94 (0.33)	0.02 (0.90)	1.70 (0.20)	3.67 (0.06)
	**rs4680_COMT**	0.89 (0.32)	2.81 (0.52)	0.73 (0.34)	1.55 (0.23)

In bold the significant effect (*p* < 0.05). * F_5, 170_ for COMT SNP and ** F_2, 34_ for COMT SNP.

## Data Availability

Data are available by request to the corresponding author.

## Supplementary Materials

The following are available online at https://www.mdpi.com/article/10.3390/genes12040555/s1, Figure S1: PVT speed across consecutive 6-h intervals of awakening according to polymorphisms of TNF-α (A), ADORA2A (B), PER3 (C), and COMT (D) in placebo (PBO) and caffeine (CAF) conditions, Table S1: ANOVA analysis of genetic polymorphism (SNPs) in interaction with awakening (TSD) and treatment (TRT) on PVT (psychomotor vigilance task) parameters (number of lapses and speed) and KSS score., Table S2: ANOVA analysis of genetic polymorphism (SNPs) in interaction with awakening (TSD) and treatment (TRT) on theta/alpha ratio in frontal and centrotemporal brain regions.

## References

[1] Connor J., Norton R., Ameratunga S., Robinson E., Wigmore B., Jackson R. (2001). Prevalence of driver sleepiness in a random population-based sample of car driving. Sleep.

[2] Goel N., Rao H., Durmer J.S., Dinges D.F. (2009). Neurocognitive Consequences of Sleep Deprivation. Semin. Neurol..

[3] Arnal P.J., Sauvet F., Leger D., van Beers P., Bayon V., Bougard C., Rabat A., Millet G.Y., Chennaoui M. (2015). Benefits of Sleep Extension on Sustained Attention and Sleep Pressure Before and During Total Sleep Deprivation and Recovery. Sleep.

[4] Basner M., Dinges D.F. (2011). Maximizing Sensitivity of the Psychomotor Vigilance Test (PVT) to Sleep Loss. Sleep.

[5] Van Dongen H.P., Maislin G., Mullington J.M., Dinges D.F. (2003). The Cumulative Cost of Additional Wakefulness: Dose-Response Effects on Neurobehavioral Functions and Sleep Physiology From Chronic Sleep Restriction and Total Sleep Deprivation. Sleep.

[6] Lieberman H.R., Tharion W.J., Shukitt-Hale B., Speckman K.L., Tulley R. (2002). Effects of caffeine, sleep loss, and stress on cognitive performance and mood during U.S. Navy SEAL training. Psychopharmacoly.

[7] Urry E., Landolt H.P., Meerlo P., Benca R.M., Abel T. (2014). Adenosine, Caffeine, and Performance: From Cognitive Neuroscience of Sleep to Sleep Pharmacogenetics. Sleep, Neuronal Plasticity and Brain Function.

[8] Killgore W.D.S., Rupp T.L., Grugle N.L., Reichardt R.M., Lipizzi E.L., Balkin T.J. (2008). Effects of dextroamphetamine, caffeine and modafinil on psychomotor vigilance test performance after 44 h of continuous wakefulness. J. Sleep Res..

[9] Lanini J., Galduróz J.C.F., Pompéia S. (2016). Acute personalized habitual caffeine doses improve attention and have selective effects when considering the fractionation of executive functions. Hum. Psychopharmacol. Clin. Exp..

[10] Hansen D.A., Ramakrishnan S., Satterfield B.C., Wesensten N.J., Layton M.E., Reifman J., Van Dongen H.P.A. (2019). Randomized, double-blind, placebo-controlled, crossover study of the effects of repeated-dose caffeine on neurobehavioral performance during 48 h of total sleep deprivation. Psychopharmacoly.

[11] Dager S.R., Layton M.E., Strauss W., Richards T.L., Heide A., Friedman S.D., Artru A.A., Hayes C.E., Posse S. (1999). Human brain metabolic response to caffeine and the effects of tolerance. Am. J. Psychiatry.

[12] Tkachenko O., Dinges D.F. (2018). Interindividual variability in neurobehavioral response to sleep loss: A comprehensive review. Neurosci. Biobehav. Rev..

[13] Satterfield B.C., Hinson J.M., Whitney P., Schmidt M.A., Wisor J.P., Van Dongen H.P. (2018). Catechol-O-methyltransferase (COMT) genotype affects cognitive control during total sleep deprivation. Cortex.

[14] Maire M., Reichert C., Gabel V., Viola A., Strobel W., Krebs J., Landolt H., Bachmann V., Cajochen C., Schmidt C. (2014). Sleep ability mediates individual differences in the vulnerability to sleep loss: Evidence from a PER3 polymorphism. Cortex.

[15] Lo J.C., Groeger J.A., Santhi N., Arbon E.L., Lazar A.S., Hasan S., Von Schantz M., Archer S.N., Dijk D.-J. (2012). Effects of Partial and Acute Total Sleep Deprivation on Performance across Cognitive Domains, Individuals and Circadian Phase. PLoS ONE.

[16] Satterfield B.C., Wisor J.P., Field S.A., Schmidt M.A., Van Dongen H.P. (2015). TNFα G308A polymorphism is associated with resilience to sleep deprivation-induced psychomotor vigilance performance impairment in healthy young adults. Brain Behav. Immun..

[17] Satterfield B.C., Wisor J.P., Schmidt M., Van Dongen H.P.A. (2017). Time-on-Task Effect During Sleep Deprivation in Healthy Young Adults Is Modulated by Dopamine Transporter Genotype. Sleep.

[18] Valomon A., Holst S.C., Bachmann V., Viola A.U., Schmidt C., Zürcher J., Berger W., Cajochen C., Landolt H.-P. (2014). Genetic polymorphisms of DAT1 and COMT differentially associate with actigraphy-derived sleep–wake cycles in young adults. Chronobiol. Int..

[19] Erblang M., Drogou C., Gomez-Merino D., Metlaine A., Boland A., Deleuze J.F., Thomas C., Sauvet F., Chennaoui M. (2019). The Impact of Genetic Variations in ADORA2A in the Association between Caffeine Consumption and Sleep. Genes.

[20] Rétey J.V., Adam M., Khatami R.O., Luhmann U.F., Jung H.H., Berger W., Landolt H.-P. (2007). A Genetic Variation in the Adenosine A2A Receptor Gene (ADORA2A) Contributes to Individual Sensitivity to Caffeine Effects on Sleep. Clin. Pharmacol. Ther..

[21] Childs E., Hohoff C., Deckert J., Xu K., Badner J., De Wit H. (2008). Association between ADORA2A and DRD2 Polymorphisms and Caffeine-Induced Anxiety. Neuropsychopharmacoly.

[22] Rogers P.J., Hohoff C., Heatherley S.V., Mullings E.L., Maxfield P.J., Evershed R.P., Deckert J., Nutt D.J. (2010). Association of the Anxiogenic and Alerting Effects of Caffeine with ADORA2A and ADORA1 Polymorphisms and Habitual Level of Caffeine Consumption. Neuropsychopharmacoly.

[23] Bodenmann S., Hohoff C., Freitag C., Deckert J., Rétey J.V., Bachmann V., Landolt H.-P. (2012). Polymorphisms of ADORA2A modulate psychomotor vigilance and the effects of caffeine on neurobehavioural performance and sleep EEG after sleep deprivation. Br. J. Pharmacol..

[24] Skeiky L., Brager A.J., Satterfield B.C., Petrovick M., Balkin T.J., Capaldi V.F., Ratcliffe R.H., A. Van Dongen H.P., Hansen D.A. (2020). TNFα G308A genotype, resilience to sleep deprivation, and the effect of caffeine on psychomotor vigilance performance in a randomized, double-blind, placebo-controlled, crossover study. Chrono-Int..

[25] Cajochen C., Wyatt J., Czeisler C., Dijk D. (2002). Separation of circadian and wake duration-dependent modulation of EEG activation during wakefulness. Neuroscience.

[26] Zigmond A., Philip Snaith R. (1983). The hospital anxiety and depression scale. Acta Psychiatr. Scand..

[27] Johns M.W. (1991). A New Method for Measuring Daytime Sleepiness: The Epworth Sleepiness Scale. Sleep.

[28] Buysse D.J., Reynolds C.F., Monk T.H., Berman S.R., Kupfer D.J. (1989). The Pittsburgh sleep quality index: A new instrument for psychiatric practice and research. Psychiatry Res..

[29] Horne J.A., Ostberg O. (1976). A self-assessment questionnaire to determine morningness-eveningness in human circadian rhythms. Int. J. Chronobiol..

[30] McLellan T.M., Caldwell J.A., Lieberman H.R. (2016). A review of caffeine’s effects on cognitive, physical and occupational performance. Neurosci. Biobehav. Rev..

[31] Khitrov M.Y., Laxminarayan S., Thorsley D., Ramakrishnan S., Rajaraman S., Wesensten N.J., Reifman J. (2014). PC-PVT: A platform for psychomotor vigilance task testing, analysis, and prediction. Behav. Res. Methods.

[32] Åkerstedt T., Anund A., Axelsson J., Kecklund G. (2014). Subjective sleepiness is a sensitive indicator of insufficient sleep and impaired waking function. J. Sleep Res..

[33] Oostenveld R., Fries P., Maris E., Schoffelen J.-M. (2010). FieldTrip: Open Source Software for Advanced Analysis of MEG, EEG, and Invasive Electrophysiological Data. Comput. Intell. Neurosci..

[34] Drogou C., Sauvet F., Erblang M., Detemmerman L., Derbois C., Erkel M.C., Boland A., Deleuze J.F., Gomez-Merino D., Chennaoui M. (2020). Genotyping on blood and buccal cells using loop-mediated isothermal amplification in healthy humans. Biotechnol. Rep..

[35] Thatcher R.W., Biver C.J., North D.M. (2004–2007). Z Score EEG Biofeedback: Technical Foundations.

[36] Landolt H.-P., Rétey J.V., Tönz K., Gottselig J.M., Khatami R., Buckelmüller I., Peter Achermann P. (2004). Caffeine Attenuates Waking and Sleep Electroencephalographic Markers of Sleep Homeostasis in Humans. Neuropsychopharmacoly.

[37] Rétey J.V., Adam M., Gottselig J.M., Khatami R., Dürr R., Achermann P., Landolt H.-P. (2006). Adenosinergic Mechanisms Contribute to Individual Differences in Sleep Deprivation-Induced Changes in Neurobehavioral Function and Brain Rhythmic Activity. J. Neurosci..

[38] Gorgoni M., Ferlazzo F., Ferrara M., Moroni F., D’Atri A., Fanelli S., Torriglia I.G., Lauri G., Marzano C., Rossini P.M. (2014). Topographic electroencephalogram changes associated with psychomotor vigilance task performance after sleep deprivation. Sleep Med..

[39] Holst S.C., Bersagliere A., Bachmann V., Berger W., Achermann P., Landolt H.-P. (2014). Dopaminergic Role in Regulating Neurophysiological Markers of Sleep Homeostasis in Humans. J. Neurosci..

[40] Wilson A.G., Symons J.A., McDowell T.L., McDevitt H.O., Duff G.W. (1997). Effects of a polymorphism in the human tumor necrosis factor promoter on transcriptional activation. Proc. Natl. Acad. Sci. USA.

[41] Louis E., Franchimont D., Piron A., Gevaert Y., Schaaf-Lafontaine N., Roland S., Mahieu P., Malaise M., De Groote D., Belaiche J. (1998). Tumour necrosis factor (TNF) gene polymorphism influences TNF-α production in lipopolysaccharide (LPS)-stimulated whole blood cell culture in healthy humans. Clin. Exp. Immunol..

[42] Jewett K.A., Krueger J.M. (2012). Humoral Sleep Regulation; Interleukin-1 and Tumor Necrosis Factor. Vitam. Horm..

[43] Chennaoui M., Sauvet F., Drogou C., Van Beers P., Langrume C., Guillard M., Gourby B., Bourrilhon C., Florence G., Gomez-Merino D. (2011). Effect of one night of sleep loss on changes in tumor necrosis factor alpha (TNF-α) levels in healthy men. Cytokine.

[44] Rogers P.J., Heatherley S.V., Mullings E.L., Smith J.E. (2013). Faster but not smarter: Effects of caffeine and caffeine withdrawal on alertness and performance. Psychopharmacoly.

[45] Auton A., Salcedo T., Metzler J.B. (2015). The 1000 Genomes Project. Assessing Rare Variation in Complex Traits.

[46] Horrigan L.A., Kelly J.P., Connor T.J. (2004). Caffeine suppresses TNF-α production via activation of the cyclic AMP/protein kinase A pathway. Int. Immunopharmacol..

[47] Hunt R.C., Simhadri V.L., Iandoli M., Sauna Z.E., Kimchi-Sarfaty C. (2014). Exposing synonymous mutations. Trends Genet..

[48] Chennaoui M., Arnal P.J., Drogou C., Leger D., Sauvet F., Gomez-Merino D. (2017). Leukocyte Expression of Type 1 and Type 2 Purinergic Receptors and Pro-Inflammatory Cytokines during Total Sleep Deprivation and/or Sleep Extension in Healthy Subjects. Front. Neurosci..

[49] Carswell A.T., Howland K., Martinez-Gonzalez B., Baron P., Davison G. (2020). The effect of caffeine on cognitive performance is influenced by CYP1A2 but not ADORA2A genotype, yet neither genotype affects exercise performance in healthy adults. Graefe’s Arch. Clin. Exp. Ophthalmol..

[50] Archer S.N., Schmidt C., Vandewalle G., Dijk D.-J. (2018). Phenotyping of PER3 variants reveals widespread effects on circadian preference, sleep regulation, and health. Sleep Med. Rev..

[51] Liberman A.R., Halitjaha L., Ay A., Ingram K.K. (2018). Modeling Strengthens Molecular Link between Circadian Polymorphisms and Major Mood Disorders. J. Biol. Rhythm..

[52] Viola A.U., Archer S.N., James L.M., Groeger J.A., Lo J.C., Skene D.J., von Schantz M., Dijk D.-J. (2007). PER3 Polymorphism Predicts Sleep Structure and Waking Performance. Curr. Biol..

[53] Vandewalle G., Archer S.N., Wuillaume C., Balteau E., Degueldre C., Luxen A., Maquet P., Dijk D.-J. (2009). Functional Magnetic Resonance Imaging-Assessed Brain Responses during an Executive Task Depend on Interaction of Sleep Homeostasis, Circadian Phase, and PER3 Genotype. J. Neurosci..

[54] Ebisawa T., Uchiyama M., Kajimura N., Mishima K., Kamei Y., Katoh M., Watanabe T., Sekimoto M., Shibui K., Kim K. (2001). Association of structural polymorphisms in the human period3 gene with delayed sleep phase syndrome. EMBO Rep..

[55] Turco M., Biscontin A., Corrias M., Caccin L., Bano M., Chiaromanni F., Salamanca M., Mattei D., Salvoro C., Mazzotta G. (2017). Diurnal Preference, Mood and the Response to Morning Light in Relation to Polymorphisms in the Human Clock Gene PER3. Sci. Rep..

[56] Gamble K.L., Motsinger-Reif A.A., Hida A., Borsetti H.M., Servick S.V., Ciarleglio C.M., Robbins S., Hicks J., Carver K., Hamilton N. (2011). Shift Work in Nurses: Contribution of Phenotypes and Genotypes to Adaptation. PLoS ONE.

[57] Bodenmann S., Rusterholz T., Dürr R., Stoll C., Bachmann V., Geissler E., Jaggi-Schwarz K., Landolt H.-P. (2009). The Functional Val158Met Polymorphism of COMT Predicts Interindividual Differences in Brain Oscillations in Young Men. J. Neurosci..

[58] Tunbridge E.M., Narajos M., Harrison C.H., Beresford C., Cipriani A., Harrison P.J. (2019). Which Dopamine Polymorphisms Are Functional? Systematic Review and Meta-analysis of COMT, DAT, DBH, DDC, DRD1–5, MAOA, MAOB, TH, VMAT1, and VMAT2. Biol. Psychiatry.

[59] Gabryelska A., Feige B., Riemann D., Spiegelhalder K., Johann A., Białasiewicz P., Hertenstein E. (2019). Can spectral power predict subjective sleep quality in healthy individuals?. J. Sleep Res..

[60] Hertenstein E., Gabryelska A., Spiegelhalder K., Nissen C., Johann A.F., Umarova R., Riemann D., Baglioni C., Feige B. (2018). Reference Data for Polysomnography-Measured and Subjective Sleep in Healthy Adults. J. Clin. Sleep Med..

[61] Santhi N., Lazar A.S., McCabe P.J., Lo J.C., Groeger J.A., Dijk D.-J. (2016). Sex differences in the circadian regulation of sleep and waking cognition in humans. Proc. Natl. Acad. Sci. USA.

[62] Adam M., Rétey J.V., Khatami R., Landolt H.-P. (2006). Age-Related Changes in the Time Course of Vigilant Attention During 40 Hours Without Sleep in Men. Sleep.

