# Supplementary material for: Genetic Determinants of Neurobehavioral Responses to Caffeine Administration during Sleep Deprivation: A Randomized, Cross Over Study (NCT03859882)
Source: Genes (Basel). 2021 Apr 10;12(4):555. doi: 10.3390/genes12040555 (PMC8069049; doi:10.3390/genes12040555)
Supplement: Supplementary file 1 [file genes-12-00555-s001.pdf]

SUPPLEMENTARY FIGURE

Supplementary figure 1. PVT speed across consecutive 6-h intervals of awakening according to polymorphisms of TNF-α (A), ADORA2A (B), PER3 (C) and COMT (D) in placebo (PBO) and caffeine (CAF) conditions.

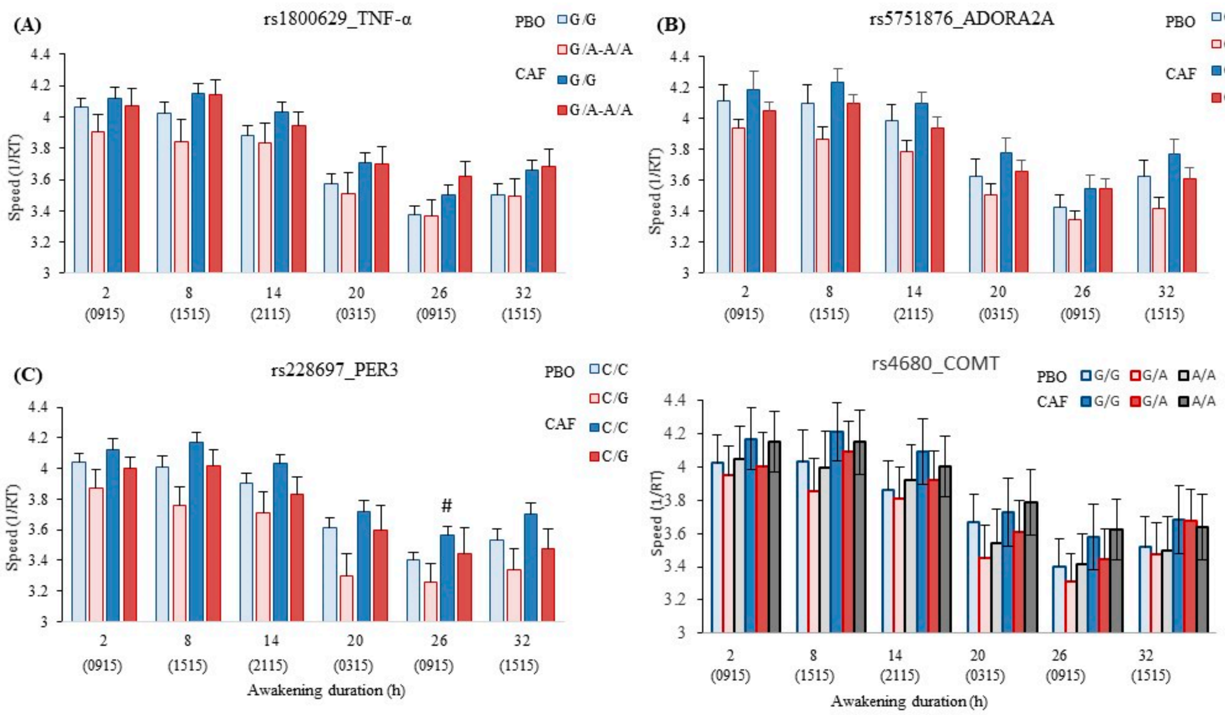

* is a SNP difference (p<0.05), # condition differences (p<0.05) for one genotype.

SUPLEMENTARY TABLES

Supplementary table 1. ANOVA analysis of genetic polymorphism (SNPs) in interaction with awakening (TSD) and treatment (TRT) on PVT (psychomotor vigilance task) parameters (number of lapses and speed) and KSS score.

Parameters	SNPs	Awakening TSD $F_{5,175}$ (p)	Treatment TRT $F_{1,35}$ (p)	Polymorphism SNP $F_{1,35}$ (p) *
**PVT Lapses**	**rs1800629_TNF-α**	**89.6 (<0.01)**	**21.3 (<0.01)**	1.40 (0.24)
	**rs5751876_ADORA2A**	**89.8 (<0.01)**	**18.5 (<0.01)**	1.45 (0.24)
	**rs228697_PER3**	**89.5 (<0.01)**	**23.5 (<0.01)**	3.06 (0.09)
	**rs4680_COMT**	**83.6 (<0.01)**	**19.8 (<0.01)**	0.04 (0.85)
**PVT speed**	**rs1800629_TNF-α**	**472 (<0.01)**	**23.6 (<0.01)**	0.19 (0.67)
	**rs5751876_ADORA2A**	**465 (<0.01)**	**23.0 (<0.01)**	2.08 (0.16)
	**rs228697_PER3**	**464 (<0.01)**	**26.5 (<0.01)**	2.54 (0.12)
	**rs4680_COMT**	**465 (<0.01)**	**22.9 (<0.01)**	0.38 (0.54)
**KSS score**	**rs1800629_TNF-α**	**215 (<0.01)**	**8.45 (<0.01)**	0.25 (0.62)
	**rs5751876_ADORA2A**	**216 (<0.01)**	**8.45 (<0.01)**	0.40 (0.53)
	**rs228697_PER3**	**217 (<0.01)**	**8.67 (<0.01)**	2.18 (0.15)
	**rs4680_COMT**	**216 (<0.01)**	**8.36 (<0.01)**	0.02 (0.89)

Bold are significant effect (p<0.05). * F2,34 for COMT SNP.

Supplementary table 2. ANOVA analysis of genetic polymorphism (SNPs) in interaction with awakening (TSD) and treatment (TRT) on theta/alpha ratio in frontal and centro-temporal brain regions.

Parameters	SNPs	Awakening TSD $F_{1,30}$ (p)	Treatment TRT $F_{1,30}$ (p)	Polymorphism SNP $F_{1,29}$ (p)*	Awakening x Treatment TSD x TRT $F_{1,30}$ (p)	Awakening x Polymorphism TSD x SNP $F_{1,29}$ (p)*	Treatment x Polymorphism TRT x SNP $F_{1,29}$ (p)*	Interaction (SNP x TSD x TRT)* $F_{1,29}$ (p
**EEG frontal**	**rs1800629_TNF-α**	3.92 (0.06)	**11.24 (<0.01)**	2.98 (0.10)	1.05 (0.31)	4.00 (0.06)	0.04 (0.84)	0.17 (0.68)
	**rs5751876_ADORA2A**	**4.02 (0.05)**	**11.31 (<0.01)**	0.16 (0.70)	1.07 (0.30)	**5.59 (0.02)**	0.23 (0.63)	0.14 (0.71)
	**rs228697_PER3**	3.73 (0.06)	**11.82 (<0.01)**	1.68 (0.21)	1.00 (0.32)	0.75 (0.39)	1.56 (0.22)	0.53 (0.47)
	**rs4680_COMT**	3.70 (0.06)	**12.09 (<0.01)**	0.14 (0.71)	0.99 (0.32)	0.31 (0.58)	2.24 (0.15)	0.46 (0.50)
**EEG centro-temporal**	**rs1800629_TNF-α**	**8.81 (<0.01)**	**9.70 (<0.01)**	1.67 (0.21)	**4.29 (0.04)**	**5.88 (0.02)**	0.11 (0.74)	0.74 (0.39)
	**rs5751876_ADORA2A**	**8.26 (<0.01)**	**10.31 (<0.01)**	0.21 (0.65)	**4.02 (0.05)**	2.32 (0.13)	1.92 (0.18)	0.26 (0.61)
	**rs228697_PER3**	**7.92 (<0.01)**	**9.99 (<0.01)**	0.93 (0.34)	3.86 (0.06)	0.00 (0.98)	0.96 (0.33)	0.05 (0.83)
	**rs4680_COMT**	**8.08 (<0.01)**	**9.72 (<0.01)**	0.25 (0.62)	3.94 (0.06)	0.68 (0.41)	0.16 (0.69)	0.57 (0.45)

Bold are significant effect (p<0.05). * F2,28 for COMT SNP.
